# A strategic approach to social accountability: Bwalo forums within the reproductive maternal and child health accountability ecosystem in Malawi

**DOI:** 10.1186/s12913-020-05394-0

**Published:** 2020-06-22

**Authors:** Nadia Butler, Ginger Johnson, Asiyati Chiweza, Kyaw Myint Aung, John Quinley, Katherine Rogers, Juliet Bedford

**Affiliations:** 1 Anthrologica, Woad Mill, Broughton, Oxfordshire, OX15 6AR UK; 2 Department of Political and Administrative Studies, Chancellor College, University of Malawi, PO Box 280, Zomba, Malawi; 3 UNICEF Malawi, PO Box 30375, Airtel Complex Area 40/31, Lilongwe 3, Malawi; 4 A Promise Renewed Secretariat, UNICEF, 3 United Nations Plaza, New York, NY 10017 USA

**Keywords:** Strategic social accountability, Maternal, newborn and child health, Political economy analysis, Community meetings, Civil society organisations, Realist evaluation, Sub-Saharan Africa, East Africa

## Abstract

**Background:**

The majority of documented social accountability initiatives to date have been ‘tactical’ in nature, employing single-tool, mostly community-based approaches. This article provides lessons from a ‘strategic’, multi-tool, multi-level social accountability project: UNICEF’s ‘Social Accountability for Every Woman Every Child’ intervention in Malawi.

**Methods:**

The project targeted the national, district and community levels. Three Civil Society Organisations (CSOs) were engaged to carry out interventions using various tools to generate evidence and political advocacy at one or more levels. This article focuses on one of the social accountability methods – the *bwalo* forum (a meeting based on a traditional Malawian method of dialogue). A detailed political economy analysis was conducted by one of the co-authors using qualitative methods including interviews and group discussions. The authors conducted in-country consultations and analysed secondary data provided by the CSOs.

**Results:**

The political economy analysis highlighted several ways in which CSO partners should modify their work plans to be more compatible with the project context. This included shifting the advocacy and support focus, as well as significantly expanding the *bwalo* forums. *Bwalos* were found to be an important platform for allowing citizens to engage with duty bearers at the community and district levels, and enabled a number of reproductive, maternal, newborn, child and adolescent health issues to be resolved at those levels. The project also enabled learning around participant responses as intermediate project outcomes.

**Conclusions:**

The project utilised various tools to gather data, elevate community voices, and facilitate engagement between citizen and state actors at the community, district and national levels. This provided the scaffolding for numerous issues to be resolved at the community or district levels, or referred to the national level. *Bwalo* forums were found to be highly effective as a space for inter-level engagement between citizens and state; however, as they were not embedded in existing local structures, their potential for sustainability and scalability was tenuous. A key strength of the project was the political economy analysis, which provided direction for partners to shape their interventions according to local and national realities and be sensitive to the barriers and drivers to positive action.

## Background

In tandem with the start of the Sustainable Development Goals (SDGs) in September 2015, the United Nations Secretary-General launched an updated version of the Global Strategy for Women’s, Children’s and Adolescents’ Health (GS 2.0). A critical priority identified in the Global Strategy was the need to promote greater accountability regarding commitments on behalf of Reproductive, Maternal, Newborn, Child and Adolescent Health (RMNCAH).^1^ Between 2016 and 2018, UNICEF^2^ implemented the ‘Social Accountability for Every Woman Every Child’ project (hereafter the Social Accountability for Health project), which focused on two of the four main actions proposed in the GS 2.0 operational framework – accountability and advocacy. The project was conducted in four high-burden focal countries: Malawi, Nigeria, Tanzania and India. The project aimed to accelerate results for women and children by strengthening national mechanisms for greater accountability around commitments to GS 2.0 and related RMNCAH targets.

This paper focuses on lessons learnt through social accountability activities carried out in Malawi as part of the Social Accountability for Health project. Malawi, a low income country with one of the highest maternal mortality ratios in the world [1], has a health system accountability landscape in which citizens, although they may be aware of their rights in terms of healthcare, have limited channels through which to voice their concerns and hold healthcare providers accountable [2]. As such, the three intended project outcomes for UNICEF Malawi were: 1) Enhanced platforms and spaces for engagement in social accountability created and deployed at the national, district and community levels; 2) Effective use of interlocutors, including media, in support of active participation and influence of policy and advocacy goals; and, 3) Evidence and recommendations generated by mapping and analysis of data and information coming from communities. These three outcomes were intended to contribute to the broader goal of improving RMNCAH results for women and children by strengthening national mechanisms for greater accountability. An initial literature review identified a lack of documentation of the process of improving health outcomes and of social accountability processes more specifically, thus our focus for this work was on process rather than outcome.

Within the last decade, the term social accountability has received increasing attention from international organisations, with numerous interventions adopting the concept, often with little interrogation into its meaning. As such, social accountability practice has tended to race ahead of relevant research and theory [3], resulting in a concept that lacks a coherent or universally accepted definition [4]. Various organisations and researchers have attempted to address this by reflecting on the meaning of the term [2–11].

In the context of this project, we have distilled a broad definition of social accountability from the various uses of the term, as follows: Social accountability refers to a range of actions and initiatives focused around a communicative relationship between citizens, civil society organisations, service providers and the government, whereby citizens organise both to hold their governments and service providers to account on pre-established norms and to influence the process of formulating new norms and service delivery outputs, usually with the support of other actors such as donors, NGOs, INGOs, the private sector, the media, and government itself. For social accountability to occur and to effect positive change for citizens, a number of enabling elements must exist, and it is largely the mandate of those implementing social accountability projects to facilitate these. We consider these to include:

1. an informed, aware and engaged citizenry;
2. a platform or platforms in which aware citizens can engage in meaningful, multi-directional communication with decision-makers and service-providers (with or without the use of interlocutors);
3. an environment in which decision-makers, having heard the concerns of citizens, respond to them, and are compelled to and have the capacity to take action.

Fox [3] promoted a useful distinction between ‘tactical’ and ‘strategic’ approaches to social accountability, where ‘tactical’ approaches are bounded, local, micro-level interventions (or tools) that are largely limited to ‘society-side’ efforts to project voice, and ‘strategic’ approaches take a macro view of the process and ‘deploy multiple tactics, encourage enabling environments for collective action for accountability and coordinate citizen voice initiatives with governmental reforms that bolster public sector responsiveness’ ([3] p8). Strategic social accountability interventions by this definition specify the multiple links in the causal chain to arrive at eventual goals, addressing areas within each of the social accountability elements described above, while the tactical approach is concerned with a specific link in the causal chain, addressing only one element of social accountability (e.g. citizen awareness) [3].

The World Bank’s Global Partnership for Social Accountability (GPSA) took the concept of strategic social accountability on board, mandating that grant applicants demonstrate how their intended social accountability interventions could be classified as strategic, and releasing a series of notes interrogating the concept and practical implications of strategic social accountability [12–17]. For the GPSA, strategic social accountability is a process that combines a set of linked, fit-for-purpose tactics, mechanisms and tools, both formal and informal, chosen on the basis of the results of a political economy analysis of the project context. In assessing more than 600 grant applications in 2013 and 2014, they found that the majority of proposals ‘did not contain the basic building blocks of a strategic social accountability intervention’ [12] and also noted, ‘Examples and evidence of strategic social accountability at work are scarce, too’ [13].

The majority of research papers to date have focused on interventions that fall under the banner of what might be termed tactical approaches, focusing mainly on community-based approaches. A number of these dealt with scorecards or report cards [18–26], and others on tools such as community meetings [27] and health facility committees [28, 29]. A small number of studies have focused on projects employing a variety of tools, although these have mostly been restricted to the local level [30, 31] or, at best, district level [32]. Roell and Mwaipopo [33] provide an example of an intervention in Tanzania that attempted to engage multiple players on both the supply and demand sides, at local and national levels. However, few papers that we are aware of have focused on multi-level interventions.

Danhoundo et al. [34] carried out a systematic literature review to identify the conditions that facilitate effective social accountability in Sub-Saharan Africa. They noted that ‘despite the increasing use of social accountability, there is limited evidence on how it has been used in the health sector.’ Only 14 studies were found that met their selection criteria.^3^ More generally, in an extensive evidence map of social, behavioural and community engagement interventions for reproductive, maternal, newborn and child health, WHO and International Initiative for Impact Evaluation [35] noted that there have been few impact evaluations of social accountability interventions, with accountability among the least mentioned outcomes in the studies identified. It was also noted that over half of the studies were from only ten countries (not including Malawi).^4^

Success in achieving the SDGs and strengthening health systems accountability will require a concerted and collaborative effort by policymakers, donors, implementers and researchers alike to generate evidence to be used to design and implement appropriate and relevant programmes and policies and to effectively measure change [36]. This article aims to contribute to the evidence base by providing lessons from a strategic, multi-tool, multi-level social accountability project. In particular, the article focuses on two aspects of the project that were found to be important: a political economy analysis carried out at the beginning of the project, and community and district *bwalos* (meetings based on a traditional Malawian method of dialogue).

We take a ‘realist’ approach, informed by the consideration of context, mechanisms and outcomes [37], where ‘mechanisms’ are non-observable, underlying processes such as ‘the cognitive, pragmatic, emotional responses’ of key actors involved in social accountability initiatives ([38] p6, [39]), which in turn lead to observable outcomes such as changed behavior. There is an increasing acknowledgement of the importance of ‘health systems ‘software’, such as norms, values and power in shaping health service delivery’ ([39] p848, [40–42]) and an understanding that providers, patients and other actors ‘exercise agency according to their reasoning and resources’ ([39] p850, [43]).^5^ It is these elements that can be positively or negatively affected by social accountability interventions.

Finally, we understand health systems to be complex adaptive systems characterised by interdependent relations that ‘reflect and enact dynamics of social and political power’ ([39] p850) and consider that social accountability occurs within an ‘accountability ecosystem’ [2, 41, 45], which is a governance landscape made up of state and non-state social actors, institutions, processes and contextual factors, as well as the relationships between these elements [41]. A growing evidence base suggests that to be effective, social accountability initiatives need to be based on an understanding of this ecosystem and to work within it, rather than attempt to work parallel to or ignore it [2, 14, 46, 47].

## Methods

Social accountability initiatives have tended to focus on feedback mechanisms and evidence gathering, but failed to engage with decision-making structures or ensure local voices reached those in a position to effect change (c.f [26, 48]). There is often an assumption in project designs that data collection and awareness-raising at the local level will somehow automatically lead to citizens demanding better service delivery, and service providers responding to those demands [14, 49]. However, there are few examples in the literature of local level social accountability efforts resulting in change at the national level, and vice versa [39]. In light of this, UNICEF Malawi employed a multi-faceted approach to social accountability, incorporating elements that targeted the national, district and community levels.

A detailed political economy analysis was conducted by one of the co-authors of this manuscript during the early phase of the project, using qualitative methods such as interviews and group discussions.^6^ Discussion guides developed for this purpose are included as an appendix. The aim of the political economy analysis was to provide a better understanding of the context into which the social accountability interventions were being introduced, including the relevant structures, actors, processes, rules of engagement, normative values, and potential barriers and drivers to positive change, to allow a flexible ‘best-fit’ approach to be adopted [50]. The resulting report [50] provided an assessment of the key laws and policies regulating RMNCAH, the strengths and weaknesses of national and district institutional frameworks for RMNCAH, the strengths and weaknesses of existing social accountability initiatives, and key information on target stakeholders, their interests, incentives, power dynamics and influence. The report highlighted contextual issues that Social Accountability for Health partners needed to be aware of in order to design interventions that were sensitive to the local context and to more effectively implement their intervention strategies, and included recommendations for CSO work plans.

Three Civil Society Organisations (CSOs) were engaged to carry out interventions within their realm of expertise that were designed to generate evidence and engender political advocacy at one or more levels (see Table 1). Their work plans were informed by the results of the political economy analysis. Parent and Child Health Initiative (PACHI) employed a number of tools, including Maternal, Newborn and Child Health (MNCH) dashboards, Quality of Institutional Care (QUIC) survey assessments and community and district *bwalos*. Youth Net and Counselling (YONECO) convened Radio Listening Clubs and Theatre for Development at the community level, and broadcast RMNCAH content on their national radio station three times a week. Malawi Health Equity Network (MHEN) carried out health budget analysis at the district and national levels, conducted health budget tracking and training at the district level, and held meetings with the Parliamentary Health Committee, Cabinet Ministers and other national-level stakeholders. MHEN also chaired a national-level CSO Task Force (the National Civil Society Health Platform on Social Accountability, or NACIPLSA), of which PACHI and YONECO were members. The project had wide geographic coverage, and was implemented across five districts: Nkhata Bay (Northern Region); Dedza and Dowa (Central Region); and Zomba and Machinga (Southern Region).

**Table 1:** CSO intervention strategies

**Organisation**	**Tool**	**Level**	**Features**	**Purpose**
**PACHI**	MNCH dashboards	Health facility	• Utilised existing Health Management Information System (HMIS) data	Data visualisation
			• Data collected by District Implementation Teams (DITs)	
			• Data validated by District Health Management Teams (DHMTs)	
			• Data packaged into ‘dashboards’	
	QUIC survey assessments	Health facility	• Conducted at Basic Emergency Obstetric and Newborn Care (BEmONC) facilities	• Data collection to assess facility readiness
				• Data visualisation
			• Data collected by DITs	
			• Data validated by DHMTs	
			• Data packaged into QUIC ‘scorecards’	
	Community bwalo	Community	• Meeting based on traditional method of dialogue	Citizen feedback and dialogue (linking citizens to health facility)
			• Held quarterly	
			• Interface between citizens and health facility	
	District bwalo	District	• Meeting based on traditional method of dialogue	Citizen feedback and dialogue (linking citizens to district-level representatives)
			• Held per month or per quarter	
			• Interface between citizens and district-level officials	
	CSO Task Force (member)	National	(see below)	(see below)
**YONECO**	Radio Listening Club	Community	• 10 per district	Citizen empowerment
			• Contributed programming to YONECO FM (YFM)	
	Theatre for Development	Community	• Community trained by Cultural Theatre Troupe	Citizen empowerment
			• Edutainment through drama	
	YONECO Radio (YFM)	National	• RMNCAH content broadcast three times per week	Data dissemination
			• Segments called ‘The Meeting Point on Health’	
			• Broadcasts conducted in local languages, Chichewa and Tumbuka	
	CSO Task Force (member)	National	(see below)	(see below)
**MHEN**	Health budget analysis, tracking and training	District	• Trained councillors, CSO representatives, DHMTs	• Increase budget transparency
				• Inform District Implementation Plan process
	Health budget analysis	National	• Desk review of 2017 health budget	Analyse for RMNCAH financial gaps
	Meetings with Parliamentary Health Committee, cabinet ministers, etc.	National	• Monthly, quarterly and annual meetings	• Highlight key findings from budget analysis
				• RMNCAH advocacy using project data
	CSO Task Force (Chair)	National	• Developed Terms of Reference (ToRs) for engaging citizens	National-level platform for social accountability stakeholders to share experiences and lessons learned
			• Six meetings held per year (one every 2 months)	
			• Multiple INGOs and CSOs included in proceedings	

*Abbreviations*: *BEmONC* Basic Emergency Obstetric and Newborn Care, *CSO* Civil Society Organisation, *DHMT* District Health Management Team, *DIT* District Implementation Team, *HMIS* Health Management Information System, *INGO* International Non-Governmental Organisation, *MHEN* Malawi Health Equity Network, *MNCH* Maternal, Newborn and Child Health, *PACHI* Parent and Child Health Initiative, *QUIC* Quality Of Institutional Care, *RMNCAH* Reproductive, Maternal, Newborn, Child and Adolescent Health, *ToR* Terms of Reference, *YFM* YONECO FM, *YONECO* Youth Net and Counselling

Community *bwalos*^7^ were held either monthly or quarterly throughout the project, providing a forum where citizens had the opportunity to voice issues of concern, receive information from district and national-level social accountability activities (packaged by PACHI in fact sheets suitable for low literacy audiences), and engage with their health service providers and local politicians. In each project site, the first *bwalo* meeting was designed to generate discussion on RMNCAH topics of concern for local communities, and the second was for PACHI and local health officials to share evidence from QUIC assessments. Issues that were not able to be addressed at the community level were referred to the respective district *bwalo*.

Similar in structure to community *bwalos*, quarterly district *bwalos* provided citizens with the opportunity to engage in discussions with district government political and administrative actors such as members of parliament, councillors, and directors (e.g. district health officer, director of planning and development). Issues raised at the district *bwalo* that were not able to be resolved at this level were referred to national platforms. By the end of 2017, 47 community *bwalos* and five district *bwalos* had been created across the five project districts.

The project was designed with a recognition of the value of adaptive learning for achieving more effective implementation of current and future social accountability projects. In light of this, Anthrologica were engaged by UNICEF to carry out learning activities to document and understand how and why particular approaches had promise or presented challenges. While this paper focuses on the *bwalo* forum, Anthrologica took a bird’s eye view of all project activities. Anthrologica carried out consultations with key governmental agencies, (INGOs) and CSOs, citizen groups, academic institutions and individual consultants, and reviewed the analyses of primary data produced by the CSOs and the political economy analysis. A topic guide and interview framework developed for this project are included as an appendix. These qualitative consultations and reviews of secondary data produced by the CSOs and the political economy analysis enabled Anthrologica to draw out lessons learnt from the various activities. These were compiled into a report for UNICEF and form the basis of this paper.^8^ The primary data collected by the three CSOs, which was intended to inform their own programming, included: documentation of citizen hearings, task force meetings and team workshops; QUIC assessments conducted at Basic Emergency Obstetric and Newborn Care (BEmONC) facilities in the five project districts; recordings of discussions and interviews by Radio Listening Clubs; sourcing of information from the community by Cultural Theatre Troupes; and documentation of community and district *bwalo* forums.^9^

## Results

### Political economy analysis and responses of project participants

The political economy analysis resulted in five key findings: 1) Malawi has, on paper, a conducive legal framework for social accountability; 2) Reproductive health policies and other guidelines emphasise accountability and citizen participation; 3) RMNCAH-related national strategies identify social accountability as an important element; 4) There has been an incomplete decentralisation process, with local government systems and structures not fully developed to ensure meaningful citizen participation or oversight of service delivery, and 5) Existing social accountability initiatives are mainly driven by CSOs focusing on community-level mobilisation, with many of them operating independently, duplicating efforts, and with minimal engagement with decision-making structures and actors above health facility level.

More specifically, the political economy analysis highlighted several ways in which the CSO partners working on the Social Accountability for Health project should modify their work plans and theory of change to be more compatible with the specific context identified through the analysis. Firstly, the political economy analysis revealed that most social accountability initiatives do not go beyond interactions at the Village Development Committee (VDC) or Area Development Committee (ADC) levels. Through the project, the interventions of the CSOs at district and national levels aimed to address this gap by elevating community voices. The analysis identified councillors who were members of the Health and Environment Committee (a committee of the district council) as key ‘interlocutors for channelling citizen voice into the policy space’ (Chiweza A: Political economy analysis of accountability for reproductive, maternal, newborn and adolescent health (RMNCAH), unpublished report), and suggested that project activities should therefore target that group of councillors and support them to make their participation in social accountability activities effective and sustainable.

On this point, one of the primary findings of a health budget tracking and training session was the need for additional capacity-building exercises for councillors, who tend to have low levels of education, as well as being new to their positions in Malawi’s recently decentralised system of local governance. Additional time was required to build this group’s skills and knowledge about health budgeting, as well as to facilitate their involvement in community and district forums, and to support them to further influence council representatives. Until the Social Accountability for Health project, most of MHEN’s activities had been related to lobbying and advocating high-level parliamentarians and national figures, an area in which they had achieved notable success. Training councillors on health budget analysis was not standard procedure for MHEN, and the organisation initially had reservations about working with weaker government actors. However, as a response to the recommendations of the political economy analysis, the engagement of councillors was incorporated into MHEN’s project work plan, with ‘mentoring’ sessions planned in addition to budget tracking and training sessions. The reorientation of project activities to better engage district councillors was a major achievement of the political economy analysis.

Secondly, the report emphasised that *bwalo* forums should be supported and significantly expanded as platforms of engagement that allow citizen groups to engage with duty bearers at the community and district levels. PACHI therefore expanded their *bwalo* forums from the two initial districts to all five project districts, and revised their concept of *bwalos* to create space for YONECO and MHEN to effectively engage with forum members. PACHI was initially unsure how to expand their operations to districts where they had few resources; however, they were responsive to the key findings of the political economy analysis, and were able to refine their strategy accordingly. As a PACHI representative concluded, ‘the refinement of our approach became more clear after the political economy analysis. Initially the focus was on the District Executive Committee and the DHMT, but we were able to shift to the decision-makers and those who are supposed to demand social accountability for health’.

As a response to the report’s recommendations, a plan was devised for YONECO Radio Listening Clubs to participate in the *bwalo* forums and use the resulting information for their programming, and for MHEN to provide a district-level analysis of health budgets in order to share information and increase transparency on health financing. It was thought that the collaborative work of all three CSOs at the district level could be an important link to establish connections across the multiple levels of influence. MHEN and YONECO were initially reluctant to meaningfully engage with *bwalo* forums, viewing these as being outside their scope of work. In order to garner the commitment and full participation of all three CSOs in the *bwalo* forums, UNICEF re-positioned the activity as a collaborative effort central to the project, and made the roles and responsibilities of each CSO explicit.

Thirdly, the political economy analysis recommended the effective use of media at all levels of influence, outlining the media’s potential to actively participate and influence social accountability at community, district and national levels. Through their ongoing work, YONECO provided multiple avenues for such engagement. However, their lack of a clearly articulated theory of change at the outset of the project contributed to a lack of responsiveness among partner CSOs to effectively use YONECO’s media reach and influence. Meanwhile, MHEN, who had a theory of change rooted in engaging high-level politicians in dialogue in order to enact change, was hesitant to collaborate with YONECO for fear that they would share information publicly without ‘first giving the government a chance to respond’. Instead, MHEN continued to work with the journalists and media houses they had established relationships with, and whom they trusted to appropriately convey messages to the public after receiving governmental approval.

Fourthly, it was highlighted that whilst there was a multitude of actors working on social accountability for health in Malawi, there was no national-level platform where stakeholders could share their experiences and lessons learned. As a result of the Social Accountability for Health project, initial steps towards establishing the National RMNCAH Social Accountability CSO Task Force were taken by UNICEF and USAID in May 2016.

The political economy consultant monitored the work of each CSO over the first phases of the project to help align their work plans and theory of change to effectively address the findings of the analysis. This process took substantially longer than initially anticipated in the project’s planning. There was a need to create space for CSO partners to collaborate, find ways of making effective use of key political and media interlocutors, and build CSO capacity in strategic areas of interest. By July 2017, CSO work plans had become synchronised and the CSOs were working together more effectively.

### Bwalo forums as an effective space for inter-level engagement

The structure of the *bwalo* forums was specific to the context and, particularly with regards to the emphasis on the involvement of community-level actors and councilors (who are key decision-makers in the district local government bodies), informed by the political economy analysis (see Table 2). Any efforts to carry out a similar intervention in another setting would require a thorough investigation of the context and key players to be included in the forum. The structure was deliberate to ensure the same social accountability evidence and advocacy efforts flowed in both directions, from the community up to the district and national levels, and from the national level down to the district and community.

**Table 2:** *Bwalo* structure and composition

**Community**	**District**
Media	Media
2–3 youth representatives (e.g. peer educators)	2–3 youth representatives (e.g. youth office)
2 persons of influence	2 persons of influence
2 community activists	2 community activists
2 religious leaders	2 religious leaders
1 faith-based organisation representative	1 faith-based organisation representative
1 CSO executive representative	5 CSO executive representatives
1 VDC representative	–
1 ADC representative	1 ADC representative
1 health advisory committee representative	5 health advisory committee representatives (BEmONC facilities)
Councillors	Councillors (Health and Environment Committee)
Traditional authorities	Traditional authorities
1 community development assistant	1 community development officer
1 school teacher	1 district education manager
1 health centre representative	District health officers
	1 social welfare representative
	1 director of planning and development
	1 member of parliament

*Abbreviations: ADC* Area Development Committee, *BEmONC* Basic Emergency Obstetric and Newborn Care, *CSO* Civil Society Organisation, *VDC* Village Development Committee

Communities across the five project districts embraced the *bwalo* forums, proving highly articulate when provided with a safe space through which to voice their concerns. Having a personal story to tell about an RMNCAH issue that had affected them directly was found to be the greatest facilitator to community participation in social accountability interventions across the project, including the *bwalos*. As an example, in a community *bwalo* in Dowa District, a close friend of a woman who died in childbirth spoke at a gathering of over 50 people, including local politicians and the media, to attest to how the health system had failed to care for her friend. The woman’s narrative led to participants offering several, sometimes competing, explanations of why the situation had arisen, and debating the mutual responsibility of health providers and communities to ensure healthy pregnancies and safe deliveries. The *bwalo* concluded with the Chair seeking suggestions about how they could ensure mothers arrive at a health facility on time, including the creation of by-laws that both community members and health officials must abide by.

*Bwalo* participants generally perceived the *bwalo*, and in particular the district *bwalo*, to be an effective method to engage directly with higher-level district officials on issues they felt had been neglected by politicians in the past. As one community *bwalo* member concluded, ‘it is difficult for communities to interact with duty bearers on our issues … through the *bwalo*, the issues get presented faster’. Although PACHI had originally intended to support one community *bwalo* per month or per quarter, some particularly active *bwalos* independently organised themselves to meet twice a month, a positive validation of the forums.

Table 3 (below) summarises key RMNCAH issues raised at community-level *bwalos* and whether they were resolved at the community or district level or referred to the national level (e.g. NACIPLSA). Most issues that were passed on to the national level were structural and related to health system strengthening needs, including staff shortages, drug thefts and stockouts, the weak referral system and the need for improved facility infrastructure (e.g. electricity).

**Table 3:** RMNCAH issues raised at community-level *bwalos* and their resolution or referral to a higher level

		**District**			
		**Dowa**	**Zomba**	**Nkhata Bay**	**Dedza**
**Issues identified**	Poor referral system	✓	✓	✓	✓
	Poor access to RMNCAH services	✓	✓	✓	✓
	Poor WASH facilities	✓	✓	✓	✓
	Inadequate staff	✓	✓	✓	
	Inadequate or no supply of electricity	✓	✓	✓	
	Harmful cultural/religious practices^a^	✓			✓
	Low capacity of local government	✓			
	Chronic shortage of drugs / supplies			✓	✓
	Limited space in health facilities			✓	✓
	No. of issues identified	39	32	16	20
	No. of issues resolved at community level	11	6	3	4
	No. of issues resolved at district level	14	11	4	4
	No. of issues referred to national level	5	5	5	5

*Abbreviations*: *RMNCAH* Reproductive, Maternal, Newborn, Child and Adolescent Health, *WASH* Water, Sanitation and Hygiene

^a^ Issues identified as ‘harmful cultural/religious practices’ were locally specific but included topics such as child marriage and adolescent pregnancy

### Intermediate project outcomes

The complexity of social accountability is such that it does not necessarily lead directly to visible outcomes in health service delivery [38]. Rather, it is key stakeholders’ responses to social accountability ‘incentives, resources or persuasion strategies provided by citizen groups that triggers change’ ([38] p3). It is therefore important to acknowledge the responsiveness and receptivity of key actors as intermediate steps in the social accountability causal chain [26, 38, 49, 51], or ‘mechanisms’ in realist terms.

Although intermediate outcomes were documented across the entire Social Accountability for Health project and its activities, these were particularly important in relation to the *bwalo* forums – an entirely new structure within the five project districts – and provided learning in relation to both community and health provider responsiveness to the forums’ purpose and mission. A number of overarching themes emerged that related to the behaviour of project participants, which were indicative of both enablers and barriers to effective engagement in social accountability strategies.

Enablers for positive engagement included motivation to act through sharing personal experiences (community members); desire to increase capacity and competency for effective budgetary oversight (councillors); recognition that evidence-based advocacy is needed for the government to increase district health budgets (health providers); and willingness of multiple national-level stakeholders to align in one forum for collective action (INGOs, CSOs). PACHI noted that participants of community-level *bwalos* were enthusiastic about their enhanced knowledge of RMNCAH issues and the health system more broadly, and were galvanised by their direct interactions with duty bearers whom they could engage on health-related matters. Local and district health authorities varied in their initial attitudes towards *bwalos*, but most ultimately came to accept them as useful platforms through which community concerns could be voiced and heard. The willingness of the various parties to participate in these fora can in itself be considered an intermediate improvement in Malawi’s social accountability environment for health.

Barriers included frustration associated with sharing personal experiences and time without seeing real change (community members); frustration with being asked to contribute time and resources that were not matched by corresponding government inputs (community members); frustration at not being provided with the adequate resources to fulfil roles and responsibilities (councillors); and fear of reprisal when speaking out against the government (health providers).

### Overall project outcomes

UNICEF has calculated that of all the RMNCAH issues raised through the various community-level tools employed by the project, approximately half were addressed at the community or district level. The types of issues addressed included: poor referral systems and lack of emergency transport equipment and systems; inadequate staff and attendance at health centers and negligent or unfriendly workers; lack of ‘youth friendly’ health services, clinics for children under five, and functional maternity wards; shortages of drugs and supplies and the suspicion that health workers divert or sell ‘free’ drugs; lack of electricity or adequate space in health centers; poor water and sanitation in health facilities; issues related to traditional customs and beliefs (e.g. child marriage, home deliveries); gender-based violence and lack of male involvement in RMNCAH; and, lack of health budget experience and training for newly appointed councillors. In reaction, district *bwalo* representatives traveled to Lilongwe in late 2017 to present issues to the national level that could not be resolved by the districts. These included inadequate drug budgets, staff and facilities, poor emergency transport and drug theft. The resolution of these issues is ongoing, but there have been commitments made by central government on some of them.

## Discussion

### Strategic social accountability – harnessing the context and engaging at multiple levels

The Social Accountability for Health project attempted to engage strategically with the RMNCAH accountability ecosystem by studying it and adapting project plans in light of knowledge gleaned from the political economy analysis, and by implementing a multi-pronged and multi-level approach to reach and effect change at different levels of the ecosystem. The project incorporated all four components promoted by the GPSA [52] as essential for strategic social accountability^10^: a) it ‘harnessed the context’ by carrying out a political economy analysis early during the project and adapting in light of the results of the analysis; b) it was ‘responsive’ in light of the recommendations of the analysis and ongoing learning, and ‘multi-pronged’ in that it utilised various tools at different levels within the ecosystem; c) it chose partners based on their reputation, credibility, unique skillset and representative legitimacy in Malawi as advocates for women and children’s health; and, d) it employed ‘active learning’ by adjusting its strategy and partners’ work plans in light of the political economy analysis and ongoing learning throughout the project.

The project’s multi-pronged nature enabled it to work to facilitate the different essential elements required for effective social accountability, as identified in the definition of social accountability we presented at the outset of this paper. Table 4 illustrates which social accountability element each of the project’s activities targeted.

**Table 4:** Social accountability elements targeted by project activities

**Element required for effective social accountability**	**Activities**
An informed, aware and engaged citizenry	• Data collected at health facility level
	• HMIS data and data from QUIC assessments packaged into dashboards, colour-coded scorecards and fact sheets in local languages to be shared with citizens at community *bwalos*
	• Radio Listening Clubs
	• Broadcast of RMNCAH content 3 times per week on YFM
	• Training of the community by Cultural Theatre Troupe
	• Edutainment theatre performances
	• ToRs developed by national CSO task force for engaging citizens
A platform/platforms in which aware citizens can engage in meaningful, multi-directional communication with decision-makers and service-providers	• Community *bwalos* linking citizens to health facilities
	• District *bwalos* linking citizens to district-level officials
	• Radio Listening Clubs contributing to programming for YFM (although unclear whether relevant decision-makers listened to these programmes)
An environment in which decision-makers, having heard the concerns of citizens, respond to them, and are compelled to and have the capacity to take action	• Councillors and DHMTs trained on health budget analysis and tracking
	• Dashboards, scorecards and health budget analysis assisted district teams to determine their performance on key RMNCAH indicators
	• Monthly, quarterly and annual advocacy meetings held with parliamentary health committee, cabinet ministers and other key players
	• Community and district *bwalos* provided decision-makers with an opportunity to engage with citizens and service providers, and to hear and respond to their concerns
	• CSO Task Force mapped social accountability activities and provided a framework for coordinating efforts at the district and national levels

*Abbreviations*: *CSO* Civil Society Organisation, *DHMT* District Health Management Team, *HMIS* Health Management Information System, *QUIC* Quality Of Institutional Care, *RMNCAH* Reproductive, Maternal, Newborn, Child and Adolescent Health, *ToRs* Terms of Reference, *YFM* YONECO FM

It is pertinent to note that the *bwalo* forums work on all three levels, serving to inform, engage and influence both citizens and state actors, at the same time as facilitating a platform for two-way communication between both groups. Other studies have similarly found interface meetings that facilitate bi-directional information sharing to provide an important space for community members to voice their concerns and ask questions of duty bearers, and for duty bearers to provide information to citizens and respond to citizen’s concerns in real time [18, 19, 21, 23, 24, 39].

### Contextual factors influencing participant responses

The available data does not allow for the construction of full Context-Mechanism-Outcome configurations [37] identifying causal links between specific contextual factors, specific mechanisms and specific outcomes. However, we have been able to identify some of the contextual factors that contributed to enabling the mechanisms described above (*bwalo* participant responses) to be activated through the experience of the *bwalo* forums:

1. Cultural contextual elements lent themselves to the effective operationalisation of the *bwalo* forum, which was based on a traditional method of dialogue, invoking the concept of an informal local system of discussing and dealing with community issues.
2. The existing social environment was such that both community members and duty bearers were willing to come together to participate in this forum.
3. PACHI is a trusted and well-connected organisation in the districts in which *bwalos* were created.

Some of these contextual factors resonate well with other studies, which have found important contributing contextual factors to include the willingness of political leaders to engage [10, 30, 39, 53] and organisational reputation and access to resources [39]. Note that the latter point as a contextual factor has not been closely investigated within the social accountability or health literature [39]. It is worth noting that sociopolitical contextual factors, such as type of government and level of free speech, will strongly affect the success of interventions such as the *bwalo* forum. Malawi’s status as a multiparty democracy and its relative level of free speech and human rights observance in recent years makes it a more favourable environment than some other countries for this type of intervention.

As highlighted by other authors, in some cases the contextual factors themselves can be strengthened by the mechanisms in a virtuous cycle [39].^11^ The contextual factors in the second and third points above, in particular, are likely to be affected, either positively or negatively, by participant responses to the forums. This was intuited by PACHI staff members, who were concerned that their institutional reputation, as well as key players’ willingness to participate in social accountability initiatives in the future, might be negatively affected if the *bwalos* were found not to be sustainable.

### Scale and sustainability

Although not always demonstrated in practice, the importance of understanding and working within local and national contexts is now widely accepted within the literature [34, 49]. However, a review of the literature shows that broadly (if not globally) applicable lessons are now beginning to emerge [39]. Elucidating these lessons is important with respect to the potential to scale up any social accountability initiative, whether within a national or transnational context.

The key overarching lesson to take away from this project - which is echoed in other projects - is that social accountability initiatives need to be strategic. The example of the *bwalos* alone shows that through the creation of communicative spaces at the community and district levels, alongside engagement with relevant national actors, numerous community-level issues were able to be raised and either resolved at the community or district levels, or referred to the national level. Having a strategic, multi-level approach allowed for this to occur. Further, a strategically designed project is more likely to lend itself to scaling up by already having spread its tentacles into various parts and levels of the advocacy ecosystem. Ideally, this means that its influence should precede it, so that, for example, sufficient effective work has been done at district level to enable different communities within that district to seamlessly adopt project tools. In this way, Halloran ([41] p7) writes that a strategic approach to scaling up accountability involves ‘taking on accountability challenges and arenas across the ecosystem – in contrast to the conventional understanding of ‘scaling up’ as simply replicating, or doing more of X’ (c.f [54].).

2In the context of a community scorecard project in Malawi, Wild and Harris [26] have also warned against neglecting systemic and national levels in favour of a focus on the local level, noting that while partners have had some success in addressing problems at the community level, issues such as procurement and resource allocation may be beyond the control of local actors. In the case of the Social Accountability for Health project, issues that could not be solved at the local level were systemic in nature and included staff shortages, drug thefts and stockouts, a weak referral system and inadequate infrastructure. Although there are no quick fixes to resolving such systemic issues, engaging with appropriate national-level players is a first and necessary step, and lays the foundations for the scaling up of local-level initiatives across the accountability ecosystem.

The Social Accountability for Health project was strategic in two key ways, and these can be replicated within other projects:

1. A political economy analysis was undertaken to better understand the accountability ecosystem and to learn about where and how the project could best exert influence on the health system. Where a political economy analysis has been done for a national accountability ecosystem, its results can be broadly applied to new local contexts within that ecosystem, although it will need to be augmented with a more localised study of key actors and enabling or limiting factors in each case.
2. A three-pronged approach was used, connecting the community, district and national levels using various tools considered to be appropriate in each case.

The following community, district and national level tactics targeted various elements of the social accountability whole, and brought results when implemented together as a collective strategy:

#### Community level (micro/local level)

- Elicit data from community health facilities to raise the awareness of citizens and to use as evidence in advocacy work with duty bearers
- Provide this information to citizens and duty bearers in an easily digestible format
- Create a space/platform for citizens to communicate with service providers and duty bearers (community *bwalo*)
- Capitalise on willing local actors/champions
- Utilise respected, well-connected facilitators/interlocutors (CSOs/traditional leaders, etc.)

#### District level (meso-level/ umbrella for many communities)

- Elicit data from district health facilities to raise the awareness of citizens and to use as evidence in advocacy work with duty bearers
- Create a space/platform for community members to communicate with district-level actors and to serve as an overarching forum to reach and engage with umbrella actors for many different communities (district *bwalo*)
- Train district-level actors on health budget analysis, training and tracking

#### National level (macro-level/ overarching all communities)

- Create a CSO task force or coalition, with the potential to utilise different members’ networks, connections, knowledge and expertise on a specific local area or for different partners to take on activities in localities in which they are embedded
- Utilise media (radio or other broadcasting methods) to provide information to citizens and duty bearers and to provide a platform for the voices of citizens or duty bearers, and potentially a space for communication between the two (although there is little evidence that the latter has yet occurred in practice)
- Conduct health budget analysis to inform citizens and duty bearers
- Conduct advocacy meetings with high-level influential actors

The potential to scale up project activities to different community settings is greater if the project has strategically and effectively engaged with multiple players at multiple levels, targeting the different essential elements for social accountability. Scale up is more likely to be successful if: 1) overarching mechanisms are in place, such as: a national CSO task force to oversee activities with a birdseye, omniscient view; engagement of high-level government officials who have the potential to influence such areas as policy, funding, deployment of health workers or medicines, etc.; and, engagement, through meetings and training, of district-level officials who oversee all communities in their district; and 2) attention is also paid to the local context within the overarching accountability ecosystem. In the context of Malawi, Wild and Harris [26] identified a number of prior conditions necessary for successful scale up of a community-based monitoring programme. These relate to the characteristics of the implementing CSOs, the quality or strength of local leadership, and specific community characteristics, such as the ability to work collectively. These conditions should be considered before expanding initiatives to new local communities, and where the optimum conditions do not exist, strategies developed for addressing or mitigating shortfalls [26].

One key learning with regard to the *bwalo* forums carried out as part of the Social Accountability for Health project was that although they were modelled on traditional forms of dialogue, their status as a new initiative, not embedded in an existing local structure or linked to a community health programme, meant that their potential for sustainability and scale up was tenuous (c.f [32].). Although the success of the *bwalo* forum meant that communities perceived it not as an activity bounded to a short-term project, but as a new, important and effective accountability structure that many wanted to continue, there has not been sufficient time for the *bwalo* forum to develop the internal resilience needed to continue without additional funding support. PACHI’s leaders expressed concern that if the *bwalo* forums were not supported, communities would feel ‘abandoned’, which may negatively affect their appetite to engage in social accountability efforts going forwards. PACHI also felt that if the *bwalo* structure proved unsustainable, their organisational reputation as a positive community advocate could be adversely affected. Likewise, some community members and district-level officials were hesitant to participate in the *bwalo* forums, perceiving them to be a temporary parallel system outside existing government structures.

This notwithstanding, in considering how best to link an initiative with an existing local structure or programme, it is important to be mindful of the potential for local elites to capture the process or resources. In countries such as Malawi, where demand for accountability by citizens is not always perceived positively by duty bearers, embedding programmes in government structures or initiatives has the potential to lessen the impact of a social accountability initiative, as state actors are given the role of interlocutors. In a bid for their project to be accepted or sustainable, CSOs in Malawi often prioritise working closely with government officials at the expense of citizens, who should be key drivers of social accountability.

Factors such as these need to be anticipated in the design of social accountability interventions and when assigning time and budgetary restrictions to a project of this nature. This is illustrative of significant and systemic concerns related to the funding landscape for social accountability. Many CSOs rely on donor support to expand and innovate in their drive to implement activities that can enact change, but few have longer-term unrestricted funding, and the risk for unsustainable programming is high. The dilemma of whether or how to embed initiatives in existing government structures or rely on external funding can be interrogated through a political economy analysis, whose value lies in the ability to guide CSOs as to where their initiative would best be situated given the specificities of the local context and key players. In the case of the present project, the political economy analysis found that it was preferable to remain independent while external funds were available.

### Limitations of the study

Because of the nature of social accountability, it is not always possible to perceive outcomes within a relatively short timeframe. As such, this paper has focused largely on intermediate outcomes, and in particular, participant responses.

An important learning aspect of the project was the difficulty of coordinating multiple partners on one project. It was challenging to manage responsibilities and competing priorities, which in turn lengthened the timeline for the project and at times meant delays in accessing secondary data. Further, a high staff turnover and changing staff responsibilities within the CSOs caused further delays and difficulties with information handover. Finally, due to time, budget and logistical constraints, the authors were limited in the amount of primary qualitative data they were able to collect.

## Conclusions

UNICEF’s Social Accountability for Health project in Malawi was strategic in its approach, utilising various tools to gather data, elevate community voices, and facilitate engagement between citizen and state actors at the community, district and national levels. The project employed active learning throughout the lifespan of the project, and engaged strategic partners with complementary skills, knowledge and social capital. The project worked strategically at the three levels to target and enhance the key elements required to enable social accountability – an informed, aware and engaged citizenry, platforms for engagement between citizens and decision-makers, and an environment in which decision-makers are willing and have the capacity to take action to effect change. The results were encouraging, with numerous issues raised by citizens at the community level being addressed at the community or district levels, or referred to the national level. Although issues referred to the national level are systemic and will not be resolved in a short timeframe, the project took the first steps in erecting the scaffolding for such issues to be elevated and heard by those capable of effecting change. The strategic nature of the project also provided greater potential for activities to be scaled up across the accountability ecosystem, bolstered by strategic allegiances and partnerships with government actors at various levels.

A key strength of the project was the political economy analysis conducted during the initial stages of the project, which provided background evidence and clear direction for UNICEF and the partner CSOs to shape their interventions according to the contemporary realities of the five implementation districts and to be sensitive to both the barriers and drivers to positive action. This ensured a flexible ‘best-fit’ approach could be adopted [50] which engaged influential actors who had the ability to increase social accountability for RMNCAH (e.g. Health and Environment Committee members), whilst simultaneously identifying weaker actors (e.g. councillors) and building their capacity to ensure the longer-term sustainability of social accountability investments.

The political economy analysis also identified the need to expand the *bwalo* forum aspect of the project. A new initiative based on traditional forms of dialogue and problem-solving, *bwalo* forums carried out at the community and district levels were found to be highly successful as a platform for communication between citizens and state actors. They provided a structure via which issues raised by citizens were able to be either addressed at the community level, referred to and addressed at the district level, or in the case of more systemic issues beyond the capacity of local actors to resolve, referred to the national level.

A key learning of the *bwalos* however, was that if initiatives are not embedded in community structures or ongoing government initiatives, they are unlikely to be sustainable in the long-term. Despite their success and their acceptance by the various key stakeholder groups, the fact that the *bwalos* had been introduced as a new initiative external to existing processes raised concerns for some actors, including the implementing CSO and government representatives, who felt that their involvement in the *bwalos* may put their local reputation at risk if the initiative did not continue. However, given the potential for elite capture, the political economy analysis did not identify appropriate existing structures with which to partner, and it was considered preferable to remain independent while external funding was available. The continuation and scale up of such initiatives requires long-term budgeting, and in order to achieve Every Woman Every Child goals and the Sustainable Development Goals, social accountability efforts must be bolstered by donor commitments to longer-term programming.

Finally, this project recognised the importance of documenting intermediate outcomes, and in particular the ‘mechanisms’ referred to in realist literature as ‘the cognitive, pragmatic, emotional responses’ of the participants of social accountability initiatives ([38] p6), which are the underlying and non-observable drivers to more concrete change or visible outcomes. Although intermediate outcomes were documented across the whole project and its activities, these were particularly important in relation to the *bwalo* forums, and provided learning in relation to both community and health provider responsiveness to the forums’ purpose and mission.

## Supplementary information

**Additional file 1.** UNICEF SAcc EWEC Malawi - Interview Guides.

## Data Availability

The data that support the findings of this study are held by UNICEF and/or the CSO partners that generated the data, but restrictions apply to the availability of these data, which are not publicly available. Data are however available from the authors upon reasonable request and with permission of UNICEF and/or the relevant CSO partner.

## Abbreviations

ADC: Area Development Committee
BEmONC: Basic Emergency Obstetric and Newborn Care
CSO: Civil Society Organisation
DHMT: District Health Management Team
DIT: District Implementation Team
DFID: Department for International Development (UK)
GPSA: Global Partnership for Social Accountability
GS 2.0: Global Strategy for Women’s, Children’s and Adolescents’ Health
HMIS: Health Management Information System
INGO: International Non-Governmental Organisation
MHEN: Malawi Health Equity Network
MNCH: Maternal, Newborn and Child Health
NACIPLSA: National Civil Society Health Platform on Social Accountability
NGO: Non-Governmental Organisation
PACHI: Parent and Child Health Initiative
QUIC: Quality of Institutional Care
RMNCAH: Reproductive, Maternal, Newborn, Child and Adolescent Health
SDG: Sustainable Development Goal
ToRs: Terms of Reference
UNICEF: United Nations Children’s Fund
USAID: United States Agency for International Development
VDC: Village Development Committee
WASH: Water, Sanitation and Hygiene
WHO: World Health Organization
YFM: YONECO FM
YONECO: Youth Net and Counselling

## Footnotes

1 This emphasis on accountability built on the first Global Strategy and the Secretary-General’s Every Woman Every Child initiative (EWEC), and was emphasised by A Promise Renewed (APR), a global effort to end preventable child death.

2 With funding from the Bill and Melinda Gates Foundation (BMGF).

3 Analysis of the 14 studies indicated that conditions facilitating effective social accountability included: ‘leveraging partnerships and building coalitions; being context-appropriate; integrating data and information collection and analysis; clearly defined roles, standards, and responsibilities of leaders; and meaningful citizen engagement’ [34p1]. Conversely, corruption, fear of reprisal, and limited funding were identified as major challenges to effective social accountability interventions.

4 Over half of the studies were derived from ten low- and middle-income countries, namely: Bangladesh, Brazil, China, Ghana, India, Kenya, Mexico, Pakistan, South Africa and Uganda. In a number of countries with the highest burden of child and maternal mortality, particularly in West Africa, no studies were identified [35].

5 While mechanisms should not be confused with the resources made available to programme participants by the programme itself, programme mechanisms, as distinct from mechanisms existing separately from the context of a programme, can be understood to be the result of the interaction between programme resources and the already existing mechanisms that manifest themselves as people’s reasoning, preferences, norms, etc. This interaction of resources and reasoning leads to decisions and choices, which in turn lead to programme outcomes [37, 43, 44].

6 Asiyati Chiweza, co-author of this paper, was engaged by UNICEF as an external consultant to carry out the political economy analysis.

7 *Bwalo* (literally, ‘circle’) is a traditional term in Malawi referring to both a neutral meeting space and the people that attend meetings convened in a neutral setting. The *bwalo* concept reinvents indigenous forms of dialogue to make them relevant in the contemporary world, and intends to invoke the traditional concept of an informal local system of problem-solving, discussing and dealing with community issues, at the same time as acting as a community citizen feedback mechanism.

8 Note that the authors did not collect, handle, store or analyse any of the primary data collected by the CSOs.

9 Consultations conducted by Anthrologica were done in English. Data collection for the political economy analysis was done in English and local languages. Data collection carried out by the three CSOs was done in appropriate local languages.

10 The four strategic components for social accountability listed by the GPSA [52] are: A. Strategies that harness the context; B. Strategies that are responsive and multi-pronged; C. Strategies that pick partners and allies that bolster your social accountability efforts; D. Strategies that employ active learning.

11 Some authors have acknowledged the challenges inherent in distinguishing between context and mechanism in some cases [43].
