# Supplementary material for: A strategic approach to social accountability: Bwalo forums within the reproductive maternal and child health accountability ecosystem in Malawi
Source: BMC Health Serv Res. 2020 Jun 22;20:568. doi: 10.1186/s12913-020-05394-0 (PMC7310083; doi:10.1186/s12913-020-05394-0)
Supplement: Supplementary file 1 — Additional file 1. UNICEF SAcc EWEC Malawi - Interview Guides. [file 12913_2020_5394_MOESM1_ESM.docx]

**Topic Guide (Anthrologica)**

**UNICEF Social Accountability for Every Woman Every Child - Malawi – August 2016**

*Purpose: To assess and analyse the context and social accountability gaps and barriers at community, structural and institutional levels, and understand the multiple levels of influence that affect decision-making in Malawi.*

**OUTCOME 1**

Participatory platforms (i.e. coalitions of NGOs and relevant civil society actors) will be identified, activated and supported in order to enhance public transparency and accountability with regard to progress made in fulfilling GS 2.0 and the commitments made on behalf of women and children. The project will document national decision-making processes around RMNCAH activities and will emphasise the political and social contexts that inform the emergence of activities undertaken by RMNCAH activists and advocates. Lessons learnt will offer insights into coalition building around RMNCAH policy at national and sub-national levels.

**Vulnerable groups in Malawi as related to RMNCAH activities (mapping)**

RMNCAH indicators to achieve (i.e. ‘costed’ targets and commitments)

**Existing models for community-level SAcc for health**

Existing investments at the community-level

Gaps in community-level health services (compared to standards and policy commitments)

Identification of issues that can/should be addressed at the district- or national-level

Tools and strategies for SAcc

Success stories (case study)

Lessons learned

**RMNCAH activists and advocates**

Methods of engagement

Ability to influence decision-makers

Bottlenecks (budget, policy, legislation)

Opportunities for engagement

**Social-cultural barriers and drivers of SAcc behaviour**

Political and social context

Organisational priorities and negotiation

Social relationships, decision-making continuum and agency to act

**Behaviour and social change**

Resistance/compliance with SAcc (willingness to participate)

Past experiences with SAcc (case study)

View and attitudes of national/district/community support of SAcc (need, type, level of support)

Difficulties enacting SAcc (community and institutional)

Barriers and drivers of change (acceptability, appropriateness, feasibility)

Divergence between policy and practice (e.g. know what should do, but don’t – why?)

Reporting procedures

**OUTCOME 2**

Inclusive citizen feedback mechanisms (i.e. SMS-based platforms, social media outreach) focusing on selected RMNCAH-related issues will be deployed and expanded. The project will document how these mechanisms can be used effectively for social accountability efforts through civil society, media and other channels, and will demonstrate their potential role in strengthening social accountability for health.

**Existing citizen feedback mechanisms to be deployed/expanded (i.e. MEHN, PACHI, and YONECO mechanisms)**

List of mechanisms and how they function (e.g. U-report, Mama na Mwana, Young Reporters Network)

Who created and why?

What area(s) of RMNCAH do/does mechanism target?

Current/planned role for mechanism in SAcc?

**Improving access and use of data for decision-making**

Who has access to? Civil society? Media?

Effectiveness of mechanism (e.g. data collection, analysis and translation)

Reach of mechanism (e.g. target population)

Use of data to prioritise, plan, and allocate resources

Practical suggestions (case study?)

**OUTCOME 3**

Civic society organisations/coalitions will have the knowledge, tools and messaging needed to increase their reach and impact, and to articulate a common ‘mass advocacy’ campaign for targeted healthcare spending, legislation, policy and other actions.

**Tools for evidence generation**

Community scorecards

Monitoring booklets

Citizen ‘voice and action’

Social audits

‘Action research’

Community action cycle

Community action groups

Policy analysis and review

Policy formulation

**Messaging and the feedback loop**

Area of feedback loop addressed by tools?

Overlapping interests/communication channels

Knowledge sharing and transfer: Community to district

Existing/planned platforms for information sharing by community action groups (Village Development Committees (VDCs), Area Development Committees (ADCs), Radio Listening Clubs (RLCs), etc.)

Structures for community engagement (issues and concerns)

Community to district-level mechanisms (e.g. district councils) mapping and identification of key decision-makers

Participation of communities (feedback from individuals?)

Bridges for communication (Identification of effective interlocutors and what they need for empowerment)

Providing community voices a platform for communication

Analysis of RMNCAH resources and mobilisation

Barriers/filters currently in place barring information collection and communication

Key bottlenecks/best opportunities

Knowledge sharing and transfer: District to national (Mangochi, Machinga, Dedza, Dowa, Nkhatabay)

Existing/planned platforms for information sharing

Structures for district engagement (issues and concerns)

District to state/national-level mechanisms (mapping) and identification of key decision-makers

Participation of district (feedback from district councils?)

Bridges for communication (Identification of effective interlocutors and what they need for empowerment)

Coalition building to direct national programme and priorities

Barriers/filters currently in place barring information collection and communication

Key bottlenecks/best opportunities

Knowledge sharing and transfer: National coalitions to political institutions

Existing/planned platforms for information sharing

Structures for national/coalition engagement (issues and concerns)

National to political-level mechanisms (mapping) and identification of key decision-makers

Participation of national coalitions (feedback from APR Civil Society Organisation Taskforce? Etc.)

Bridges for communication (identification of effective interlocutors and what they need for empowerment)

Barriers/filters currently in place barring information collection and communication

Key bottlenecks/best opportunities

**Sample Interview Framework – Civil society organisations (CSOs) (Anthrologica)**

**UNICEF Social Accountability for Every Woman Every Child - Malawi – August 2016**

**INTERVIEW DATA**

- Country/District/Village:
- Organisation:
- Date:
- Unique ID code:
- Time/duration of interview started:
- Name of interviewer:

- Name of back-up note taker (if used):
- Name of translator (if used):
- Digital recording code:
- General comments and observations:

**PARTICIPANT INFORMATION**

| **Sex** | **Age** | **Time in service** Years and months | **Education level**  Primary, secondary, tertiary | **Position** | **Type of organisation**  e.g. child health, reproductive and sexual health, etc. |
| --- | --- | --- | --- | --- | --- |
|  |  |  |  |  |  |

Note: *Recommend reviewing content of each interview to allow for iterations based on organisation area of expertise (e.g.* YONECO *media activism).*

**1. Background.**

- - *What is the mission and values of your organisation?*
  - *How long have you been in operation? In what areas do you operate?*
  - *In what ways is your organisation involved with RMNCAH activities (programming, strategy, policy)?*
  - *What are your organisational commitments to RMNCAH activities?*
  - *[For UNICEF contracted organisations] What programme activities is your organisation responsible for with regards to SAcc for RMNCAH?*

**2. Mapping and coordination.**

- - *Other than your organisation, what others exist that work on RMNCAH issues? List.*
  - *Have you ever worked with any of these organisations? Why or why not?*
  - *Would you like to work with any of these organisations now or in the future? Why or why not?*
  - *What successes/challenges would you foresee that may prevent you from working effectively with these organisations? Explain.*
  - *Is there joint learning and collaboration? If not, how do you think this can occur?*
  - *How is the coordination between the government, INGOs and civil society?*

**3. What kind of data/information does your organisation need in order to function most effectively for addressing SAcc as it relates to RMNCAH?**

- - *What do you need to know?*
  - *When do you need to know it?*
  - *How does this information need to be presented/formatted for understandability?*
  - *Why do you need to know this? How will knowing this help you to better address SAcc for RMNCAH?*
  - *Does this information already exist and do you need access to it? Does this information need to be collected for the SAcc project? [If relevant, ask interviewee to assign a priority rating to this data. E.g. ‘urgent’]*
  - *Who/what/where do you need to collect this information from? (age, gender, location, position, organisation, etc.)*
  - *Are there any foreseeable challenges to collecting this kind of information? Have you tried to collect this information in the past and were unsuccessful? Explain.*

**4. What (if any) citizen feedback mechanism(s) has your organisation used in the past/is planning to use/would like to use as part of the SAcc project?**

- - *What is the mechanism? What does it do? How does it function? [Generate list of mechanisms to be used in subsequent questions].*
  - *When was this mechanism first used, for what purpose and by whom?*
  - *Who has access to this mechanism? Civil society? Media? Government?*
  - *Who/what population does this mechanism reach? (age, gender, etc.) What level? (community, district, etc.)*
  - *What has been your organisation’s experience with this mechanism? Successes? Challenges?*
  - *Why do you like/want to use this mechanism? What data/information do you think this mechanism could best capture for SAcc as it relates to RMNCAH?*
  - *Is information collected by this mechanism intended to be communicated back to the community? Decision-makers? Both? How?*
  - *Do you need (additional) support in order to use this mechanism most effectively? What do you need? Explain.*
  - *Do you have any questions or concerns regarding the use of this mechanism?*

[Questions 5 and 6 can be answered from the perspective of data collection needs, on-going data collection activities, or planned data collection depending on the status of the organisation and their answers to the previous questions.]

**5. How should/is/will the data/information for _______________ mechanism be collected and analysed?**

- - *Data collection activities and challenges encountered (what, who, when, where, why, how)*
  - *Data analysis activities and challenges encountered (what, who, when, where, why, how)*

[Repeat series of questions with every mechanism listed]

**6. How should/are/will the findings from _______________ mechanism be reported and shared?**

- - *How will data be aggregated?*
  - *How will it be prioritised for planning and resource allocation?*
  - *Who is the accountably party that should receive this information? Do they have the power to act (i.e. agency) on the information shared?*
  - *How will data be shared (when, how, with whom)? What approach would you take to try and influence (communities, decision-makers) with this information?*
  - *How efficient is this method of sharing? How effective?*

[Repeat series of questions with every mechanism listed]

**7. What (if any) citizen feedback mechanism(s) would your organisation not like to use as part of the SAcc project?**

- - *What is the mechanism? What does it do? How does it function?*
  - *When was this mechanism first used, for what purpose and by whom?*
  - *Who has access to this mechanism? Civil society? Media? Government?*
  - *What has been your organisation’s experience with this mechanism?*
  - *Why do you not want to use this mechanism? Past failures of this mechanism?*
  - *What questions or concerns do you have regarding this mechanism?*

**8. Do you have anything else you would like to share with us?**

**Thank you for your time and for sharing your opinions and experiences with us.**

[RECORD STOP TIME] __________

**Political Economy Analysis – Discussion Guide for Key Stakeholders (Dr Asiyati Chiweza)**

**UNICEF Social Accountability for Every Woman Every Child - Malawi – October 2016**

The UNICEF approach to this project is on accountability and advocacy in order to consolidate and strengthen achievement of country RMNCAH targets and commitments.

1. What are the key challenges regarding social accountability for RMNCAH service delivery?
   1. What is the evidence that shows that there are significant accountability challenges with RMNCAH service delivery?
2. What are the existing institutional arrangements that promote or constrain accountability for RMNCAH service delivery and commitments?
   1. What are the key existing institutional or governance arrangements at the district and community level within which the accountability function is supposed to take place, structures for effective participation and community voice in health programs and how community feedback is supposed to reach duty bearers?
   2. What is their role, what are they accountable for? What do they focus on?
   3. What are the relationships in the accountability framework?
   4. Are the existing institutional or governance arrangements capable, effective and efficient for RMNCAH accountability?
   5. Key stakeholders are organisations, individuals or offices that have vested interests in and have influence to shape the practice of accountability in RMNCAH framework.
      1. Analysis of interests, incentives, attitudes and influence of each stakeholder
      2. Knowledge of their roles and key policies and strategies concerning RMNCAH
   6. CSOs - which ones are doing RMNCAH-related work in this district, what do they focus on, where do they operate, contact person
   7. Role of CSO Network, MOU exist
   8. What rules exist to enhance accountability?
3. What are the key political economy drivers that explain or underpin the status quo?
   1. Why are things the way they are in terms of RMNCAH accountability?
   2. Why are policies or institutional arrangements not being improved?
4. What is it that can be done to strengthen social accountability for Every Woman Every Child?
   1. What actions can be proposed in order to change the current situation?
   2. Are the proposed changes or interventions going to work within the existing reform space or should there be attempts to expand it?

**Political Economy Analysis – Discussion Guide for RMNCAH Health Centres and Communities (Dr Asiyati Chiweza)**

**UNICEF Social Accountability for Every Woman Every Child - Malawi – October 2016**

**Meeting with Health Centre Staff**

1. What are the key Reproductive, Maternal, Child and Adolescent Health (RMNCAH) issues affecting this area?
2. Describe the health centre accountability system
3. What is the role of the Health Centre Advisory Committee (HAC)?
4. Does it play any role on matters of drugs and other supplies?
5. How does it work?
6. Elaborate the strengths and weaknesses of the HACs
7. Is there any link between the HAC and Village Health Committees?
8. What role do Village Development Committees (VDCs) and Area Development Committees (ADCs) play in RMNCAH issues?
9. What role does the Health and Environment Committee of the council play in RMNCAH issues?
10. Which is the most important institution for taking RMNCAH issues from the community to the council?
11. Which is the most important institution for bringing feedback on RMNCAH issues from the council to the community?
12. Who are the most important actors in taking citizens’ concerns on RMNCAH to the council?
13. Who are the most important actors in taking citizens’ concerns on RMNCAH to the Health Office?

**Meeting with Health Centre Advisory Committee**

1. What are the key Reproductive, Maternal, Child and Adolescent Health (RMNCAH) issues affecting this area?
2. What is the role of the Health Centre Advisory Committee?
3. Does it play any role on matters of drugs and other supplies?
4. How does it work?
5. Elaborate the strengths and weaknesses of the HACs
6. Is there any link between the HAC and Village Health Committees?
7. What role do VDCs and ADCs play in RMNCAH issues?
8. Which is the most important institution for taking RMNCAH issues from the community to the council?
9. Which is the most important institution for bringing feedback on RMNCAH issues from the council to the community?
10. Who are the most important actors in taking citizens’ concerns on RMNCAH to the council?
11. Who are the most important actors in taking citizens’ concerns on RMNCAH to the Health Office?

**Meeting with VDC Members**

1. What is the role of the VDC in this area?
2. What are the key Reproductive, Maternal, Child and Adolescent Health (RMNCAH) issues affecting this area?
3. What role does the VDC play in RMNCAH issues?
4. Where do you channel RMNCAH issues affecting this village?
5. Which is the most important institution for taking RMNCAH issues from the community to the council?
6. Which is the most important institution for bringing feedback on RMNCAH issues from the council to the community?
7. Who are the most important actors in taking citizens’ concerns on RMNCAH to the council?
8. Who are the most important actors in taking citizens’ concerns on RMNCAH to the Health Office?

**Meeting with women and youth representatives (for youth, talk about adolescent health issues)**

1. What are the key Reproductive, Maternal, Child and Adolescent Health (RMNCAH) issues affecting this area?
2. Where do you channel such issues?
3. Which is the most important structure in dealing with RMNCAH issues that citizens have? Please list 3 and rank them in order of importance.
4. Which is the most important institution for taking RMNCAH issues from the community to the council?
5. Which is the most important institution for taking RMNCAH issues from the community to the Health Office?
6. Which is the most important institution for bringing feedback on RMNCAH issues from the council to the community?
7. Who are the most important actors in taking citizens’ concerns on RMNCAH to the council?
8. Who are the most important actors in taking citizens’ concerns on RMNCAH to the Health Office?
